# Supplementary material for: The relationships between children’s motor competence, physical activity, perceived motor competence, physical fitness and weight status in relation to age
Source: PLoS One. 2023 Apr 14;18(4):e0278438. doi: 10.1371/journal.pone.0278438 (PMC10104338; doi:10.1371/journal.pone.0278438)
Supplement: S1 Table — * 1,96 > z > -1,96 is significant. * n/a refers to a correlation coefficient being absent because measurements were net performed in that age group. The–means that one or two of the correlation coefficients were not significant. In both situations, no calculations on the significance of the difference could be performed. (DOCX) [file pone.0278438.s001.docx]

|  | **Age 5** | **Age 6** | **Age 7** | **Age 8** | **Age 9** | **Age 10** | **Age 11** | **Age 12+** |
| --- | --- | --- | --- | --- | --- | --- | --- | --- |
| **Age 4** | - | - | - | - | - | - | - | - |
| **Age 5** |  | - | - | - | - | - | - | - |
| **Age 6** |  |  | - | - | - | - | - | - |
| **Age 7** |  |  |  | - | - | - | - | - |
| **Age 8** |  |  |  |  | - | - | - | - |
| **Age 9** |  |  |  |  |  | -0,047882 | -0,977721 | - |
| **Age 10** |  |  |  |  |  |  | -0,89896 | - |
| **Age 11** |  |  |  |  |  |  |  | - |
