# Supplementary material for: The relationships between children’s motor competence, physical activity, perceived motor competence, physical fitness and weight status in relation to age
Source: PLoS One. 2023 Apr 14;18(4):e0278438. doi: 10.1371/journal.pone.0278438 (PMC10104338; doi:10.1371/journal.pone.0278438)
Supplement: Attachment — Submitted filename: Response to Reviewers.docx [file pone.0278438.s011.docx]

February 14, 2022, Amsterdam

Dear reviewers,

Thank you for your elaborate review of our manuscript entitled “*The relationships between children’s motor competence, physical activity, perceived motor competence, physical fitness and weight status in relation to age”.* We appreciate your compliments and comments and thereby the opportunity to increase the quality of our work. We hope our revised version satisfies your remarks.

We would hereby like to respond to the questions and suggestions in your review. When we refer to line numbers, these refer to the ‘Manuscript with Track Changes’.

## Reviewer 1

Comment 1.1
*“While the cross-sectional nature of the data limits the generalizability of the conclusions, the data generally support other longitudinal data from studies that have examined a limited number of variables in the model. It might be useful to address the results of these studies to corroborate or refute the results of the current study as the age ranges of the below studies are within the age range of the current study”.*

Thank you for these suggestions. We have added the results from the suggested longitudinal studies to our discussion to better address our results in relation to existing literature. This now reads:

“Our findings show that children in late childhood who have higher perceived motor competence are also more physically active, have higher physical fitness, higher motor competence and lower BMI. Although this does not prove causality, it does align with the proposed spiral of (dis)engagement as described by Stodden et al.[16]. In middle childhood this relationship with BMI is not fully developed yet. **Some longitudinal studies also support the proposed spiral. While motor competence influences fitness and fatness (directly and via fitness) from a young age [66,67] and perceived motor competence later in childhood [68], motor competence [69,70], perceived motor competence [68], fitness [23,69] and weight [69] all appear to influence physical activity in late childhood.** Our findings therefore reinforce the idea that focusing on motor competence in early childhood might be a feasible way to ensure continued participation in physical activities throughout childhood and adolescence and thereby reducing obesity problems.” [lines 391-400].

Thereby we have also adjusted our directions for future research as follows:
“However, our results indicate that targeting motor competence and perceived motor competence at a young age might be a feasible way to ensure continued participation in physical activities throughout childhood and adolescence. Yet, how to effectively influence motor competence is still largely unknown: both our data and available literature suggest that only increasing physical activity will not be enough [9], while maintaining a healthy weight could be a promising starting point to kick off a positive spiral [9,67]. Future research should thus aim to unravel how to improve motor competence. **In addition**, large scale longitudinal **studies** including all variables and all age groups **are necessary to gain more insight in the directions of these relationships through developmental time**, ideally while making the distinction between different aspects within each variable.” [lines 472 – 482].

Comment 1.2 *“While the individual correlations among variables at each age is important in its own right, I am left wondering if it would be possible to analyze the data at each age as a collective system (e.g., using SEM/path analyses) as it would provide understanding of whether the data collectively “fit” the Stodden et al model as a whole (i.e., as a more comprehensive system of individual factors of development).”*

You have suggested to complement our analysis with an analysis in which the model is tested as a collective system in each age category using SEM. We have carefully discussed and considered the possibilities to do so, together with our statistician. To really test the collective system that is proposed by Stodden et al. (2008), this includes both (a) the direction and (b) the strength of the relationships. However, in regards to the direction: the cross-sectional nature of our data precludes the ability to find the direction of effects by the use of a SEM-analysis. It would be possible to specify the relationships in the model in both directions, but we do not have enough degrees of freedom to do so. Moreover, this still does not proof causality. To test an increase of strength of the relationships as a collective system, as proposed in the model by Stodden et al. (2008), is unfortunately not feasible. Since there are no restrictions to the model (all variables are related, thus freely estimated), this automatically leads to a perfect fit of the model in all ages. This leads to the final analysis in our paper: since we did not include latent variables nor path analyses, this results in a correlational analysis.
We sincerely appreciate the suggestion and think it is interesting and possible for longitudinal analyses. However, for our current study, we conclude that the correlational analysis is the fair way to treat these data and hope that you can concur with us.

Comment 1.3 *“Another potential limitation of the current statistical analyses is the assumption of linear relationships between variables. The authors address this idea indirectly when referencing a proficiency barrier, but it is still a potential avenue for exploration, perhaps in a subsequent paper.”*

The remark on the assumption of linear relationships is very interesting. Since our starting point was the article by Stodden et al. (2008) and possible nonlinearity is not discussed there, we have moved forward with the assumption of linear relationships. Especially since this is commonly done in related studies on the same subject. However, based on your remark we have also found articles that propose non-linear relationships between physical activity and motor skills (Bezerra et al., 2021) and between BMI and motor coordination (Lopes et al., 2018). We visualized our data in scatterplots and checked for linearity. Although our data do not give us reason to assume nonlinear relationships, it is indeed an interesting avenue for further exploration. Especially since this is probably highly dependent of the way the variables are measured and quantified. We have therefore added this suggestion to our suggestions for future research as follows:

**“Moreover, the possibility of nonlinearity of these relationships should be further investigated, since non-linear relationships between physical activity and motor skills [80] and between BMI and motor coordination [81] have been described**.**”** [lines 482 - 485]

Comment 1.4 *While the “motor age” variable partially addresses how motor skill scores generally increase with each age group, would it be useful to provide supplementary data to see the changes in raw motor skill scores across age groups? The authors noted a potential ceiling effect for the motor skill measures; thus, it might be useful to provide the raw data to better show the how motor skill levels change across time.*

After reading this question, we fear that we have been unclear in the description of our data. We have therefore adjusted our description of the 4-Skills Test and it’s outcomes to hopefully provide a clearer explanation of the outcome scores. To address your question directly: the variable “motor age” already is the raw total score. The test consists of different levels, each level represented by an activity that children can either succeed or fail at performing. The highest activity that they succeed at performing, gives them the motor age of that level. This mean motor age per age category is included in Table 1. Our revised description is as follows:

*“The subscales contain 11 elements of increasing difficulty. Each element* ***represents*** *a ‘motor age’:* ***the age based on the depicted motor skill competence.*** *For example, 6-year old children are expected to be able* ***to skip. If a child successfully skips (and fails at subsequent elements), they score a motor age of 6.*** *The mean of the four components* ***forms*** *a total score. Comparing motor age to calendar age leads to a score for ‘motor lead’****, the final score used in our analyses****. A positive motor lead value indicates that a child performs better than to be expected based on calendar age, a negative motor lead value indicates that a child performs lower than to be expected.“* (lines 164 – 173)

We have considered adding a scatterplot that shows how calendar age and motor age relate to each other (in order to address the ceiling effect of the test). However, we think that that would go beyond the scope of the article. In addition, since Table 1 already shows the mean motor age in every age category, this already shows how motor age does not increase in parallel with calendar age from the age of 10. We hope that sufficiently answers your question.
N.B. A manuscript about the psychometric properties of the 4 Skills Test is being prepared by dr. W.G. van Kernebeek and will potentially answer follow-up questions about the 4 Skills Test in the future.

Comment 1.5 *One important limitation in the data is based on the measure of PA. I believe it is important to note the limitations of these data more concretely. Specifically, the overestimation of PA with questionnaires should be noted.*

Although we have attempted to collect our data in the most valid way possible, the indirect measure of PA is indeed an important limitation of our dataset. We believe that although overestimations of PA might occur due to the use of a questionnaire, this will not change the nature of the relations with PA. Nonetheless, since it is a limitation of our dataset, we have added this limitation to our discussion in the following way:

**“In addition, assessing physical activity by use of a questionnaire often leads to an overestimation of physical activity [73].”** [lines 446 – 447]

Comment 1.6 *Lastly, addressing maturation and how that impact gender-specific differences in the relationships (specifically during the adolescent transition) also is an important notion that was not explored in the data. Controlling for gender in the correlations might be a useful endeavor from a statistical standpoint.*

This is indeed an interesting endeavor. In earlier analyses we have explored sex-specific differences by calculating all correlations separately for boys and girls. However, with these analyses we were not able to recognize patterns in the development of relationships between variables, possibly caused by the accompanying decrease in sample size. Since our sample size did not allow us to make these subgroups and the analyses did not provide a clear addition to our results, we have not included these in the final manuscript. However, it would indeed be interesting to see if the development of the relationships was different for boys than for girls. We have therefore elaborated more on this in our suggestions for future research as follows:

“**Lastly**, addressing sex differences in these relationships might also be interesting as it has been proposed that the mediating role of perceived motor competence might be stronger for girls than for boys [26] **and that reciprocal relations between motor competence, endurance and fatness are dependent on sex [67].** **Since boys and girls do not go through their maturational stages simultaneously [82], exploring sex differences in the developmental nature of the studied interrelations would be a valuable direction for follow-up studies.”** [lines 485 – 491]

## Reviewer 2

Comment 2.1  *“Update the reference [2]”*

We realized that the study by Guthold et al. (2018) is a study that uses the WHO data and is performed by WHO employees and that the WHO does not have a new report on worldwide physical activity. We therefore conclude that the reference [1], Guthold et al. (2018), is sufficient and we have removed the WHO reference [2].

Comment 2.2  *“Give more information about the socioeconomic status of the sample or the population from which the sample was drawn.*”

We have added our available information on the SES of the participating schools, however, we do not have this data on a child-level. We have an estimation of SES based on the postal code of the school and we have added this information to our participants section as follows:

**“Based on postal codes, three schools were located in an area with low socio-economic status, two in medium and one was located in an area with high socio-economic status.”** [line 144 – 146]

Comment 2.3  *“Give more details of excluded participants (exclusion percentage, gender, age group)”*

Unfortunately, we do not have accurate data in regards to excluded participants. When parents did not consent with their child’s participation, these children were not included in the student lists. We are therefore unable to give more information on these children.

Comment 2.4  *“Give more details on how the sampling was done; explain how the sample size was estimated.”*

We have added information on sampling to our methods in the following way:

**“An a priori sample size calculation was done using Gpower 3.1 (Windows, Düsseldorf, Germany)[29]. With a power estimate of 0.80, alpha set at 0.05 and an effect size of 0.20, this led to a required sample size of 193. Taking into account expected exclusions, we aimed at including approximately 250 children per group and therefore 6 schools.”** [lines 141 – 144].

“**All children of these schools were invited to participate in the study.** Data was collected **in a final sample of** 2068 children (age 4 – 13, 50,6% boys). **In these children, at least one test was performed.** Descriptive statistics on the study sample can be found in Table 1. Reasons for exclusion were absence, injury and absence of informed consent.” [lines 151 – 153].

Comment 2.5  *“Lines 300-302: “Although we cannot draw conclusions on causality, our findings do not support the proposed pathway in which physical activity stimulates motor competence in early childhood.”
This sentence, unfortunately, is poorly constructed and may lead the reader into a misunderstanding.* *The authors are asked to consider rewriting it, bearing in mind that the data in this article preclude any stimulus/cause-effect inference. The choice of terms here must be very careful.”*

Thank you for this recommendation. We have rewritten this sentence and hope that in this way we do not imply to have investigated cause-effect relations. This now reads:

“At the center of the model by Stodden et al. [15] is the relationship between motor competence and physical activity, that changes direction from early childhood to middle childhood. **They propose that in early childhood, a weak relationship between motor competence and physical activity exists: physical activity stimulates the development of motor competence. We cannot confirm this proposed pathway, since we found no correlation between these two variables in 4- to 6-year old children.”** [lines 308 – 315]

Comment 2.6  *“It is also suggested that the authors reflect a little more on the results of physical activity and possible biases in data collection, as it is precisely at younger ages that n is the lowest for the physical activity variable.”*

This is a good point. Aside from the previously mentioned limitation in the measure of PA, we have included the potential bias and low sample sizes that resulted from sending questionnaires to the parents to the discussion. This now reads as follows:

“This leads to the next limitation of this study, which is the lower response rate for the physical activity questionnaire that was sent to the parents. Although this was expected**, it possibly led to bias in the data and** led to a significantly lower sample size for the analyses that included physical activity **in the younger children. Since it’s also in the younger children that we found no relationships with physical activity, some caution is warranted there. In addition, assessing physical activity by use of a questionnaire often leads to an overestimation of physical activity [73].”**[lines 441 – 447]

Comment 2.7  *“Line 302-305: at this point, it should be considered that the present study used indirect measures of physical activity”.*

Since this comment was also made by the first reviewer, we have addressed this limitation in the limitations section of our discussion [lines 446-447], as described in our reaction on comment 2.6. We hope that suffices for the second reviewer as well.

Thank you for receiving our revised manuscript. We appreciate your time to consider it for review and look forward to your response.

With kind regards,

Anne Rosalinde den Uil (MSc)
[a.r.den.uil@hva.nl](mailto:a.r.den.uil@hva.nl)
tel: +31 6 11418281

Amsterdam University of Applied Sciences
Centre of Expertise Urban Vitality
Faculty of Sports and Nutrition
Dr. Meurerlaan 8
1067 SM Amsterdam
The Netherlands
